# Ischemic Stroke Secondary to Myocarditis in the Setting of COVID-19

**DOI:** 10.7759/cureus.34446

**Published:** 2023-01-31

**Authors:** Tarakarama Musunuri, Patrick M Muehlberger

**Affiliations:** 1 Emergency Medicine, Brooke Army Medical Center, Fort Sam Houston, USA

**Keywords:** thrombo embolic disease, cardiomyopathy, stroke, ischemic stroke, myocardidits, covid 19

## Abstract

Coronavirus disease (COVID-19) is primarily a respiratory disease that has also been shown to be associated with neurological complications such as ischemic stroke, Guillain-Barré syndrome, and encephalitis. Ischemic stroke in patients with COVID-19 has mostly been observed in the elderly, those with significant comorbidities, and the critically ill. In this report, we discuss a case of ischemic stroke in an otherwise healthy young male patient who only had a mild case of COVID-19. It is likely that the patient suffered from an ischemic stroke secondary to cardiomyopathy that resulted from the severe acute respiratory syndrome coronavirus 2 (SARS-CoV-2) infection. The ischemic stroke was most likely a result of thromboembolism caused by stasis of blood from acute dilated cardiomyopathy and the hypercoagulable state of COVID-19 patients. It is important to maintain a high degree of clinical suspicion for thromboembolic events in COVID-19 patients.

## Introduction

Coronavirus disease 2019 (COVID-19) caused by the severe acute respiratory syndrome coronavirus 2 (SARS-CoV-2) virus has been linked to many complications and has resulted in significant mortality and morbidity. COVID-19 typically presents with mild systemic illness including fevers, cough, and shortness of breath. Less common symptoms of COVID-19 include anosmia, ageusia, ataxia, and confusion [1]. Complications associated with this disease most commonly manifest in the pulmonary and cardiac systems. Patients have been seen to develop acute respiratory distress syndrome (ARDS), pulmonary embolism, superimposed bacterial infections, pericarditis, and myocarditis [2]. However, in a subset of patients, neurological complications such as acute ischemic stroke, encephalitis, and Guillain-Barré syndrome have been demonstrated [3]. Severe COVID-19-related complications have been more commonly observed in patients with comorbidities such as obesity, hypertension, diabetes, and underlying cardiac or pulmonary disease [4]. This report presents the case of a 29-year-old male without any significant comorbidities, who presented with ischemic stroke in the setting of COVID-19 and myocarditis. The majority of acute stroke cases in the setting of COVID-19 have been demonstrated in patients over the age of 50 and those with significant comorbidities or risk factors [4].

## Case presentation

A 29-year-old active-duty senior airman presented to the Emergency Department on May 12, 2021, via ambulance for sudden onset right arm weakness and aphasia that started two hours prior to arrival. No interventions were carried out by the emergency medical service personnel en route to the hospital. Upon arrival, the patient had normal vital signs and a point-of-care glucose of 129. History was notable for COVID-19 infection on January 24, 2021. He developed fever, cough, and myalgias that lasted for approximately two to three weeks. During March and April of 2021, the patient reported worsening dyspnea, 15-pound weight loss, severe fatigue, and paroxysmal nocturnal dyspnea prior to his presenting symptoms. The patient did not have any preexisting medical conditions such as diabetes, cardiovascular disease, or thrombotic events. 

**Figure 1:**
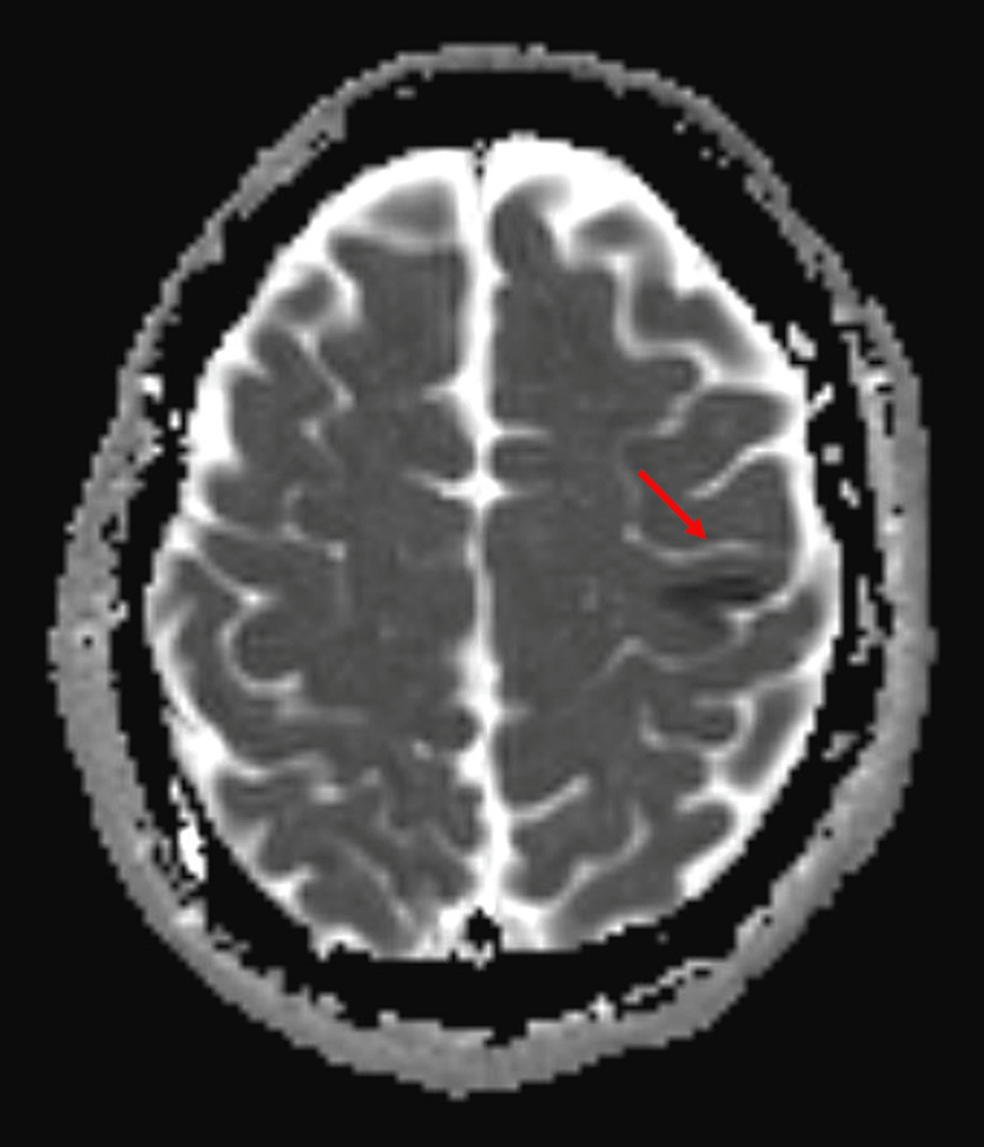
The ADC MRI image shows restricted diffusion with corresponding low ADC signal at the lateral portion of the primary motor cortex indicating hyperacute ischemic infarct (Red Arrow). ADC: apparent diffusion coefficient

Physical exam showed 2/5 strength in the right hand and significant right lower facial paresis. Given the constellation of findings, CODE STROKE was initiated. After CT imaging and evaluation by the neurology team, the decision to proceed with MRI was made. MRI confirmed a left middle cerebral artery stroke, as shown in Figure 1. The patient was given tissue plasminogen activator (tPA) with improvement in his presenting symptoms of right upper extremity weakness and facial asymmetry. The patient was subsequently admitted to the ICU for continued care.

Further evaluation as to why the patient had a stroke included an echocardiogram of the heart and a CT angiogram of the chest. During the stroke workup, he was found to have segmental pulmonary emboli and left basilic vein nonocclusive thrombus. Cardiac MRI, transesophageal echocardiogram, and heart catheterization established significantly newly diagnosed dilated cardiomyopathy with a severely reduced left ventricular ejection fraction of 20%. He was subsequently started on a heparin drip and goal-directed medical therapy for his heart failure, which included lisinopril, metoprolol, and empagliflozin.

**Figure 2:**
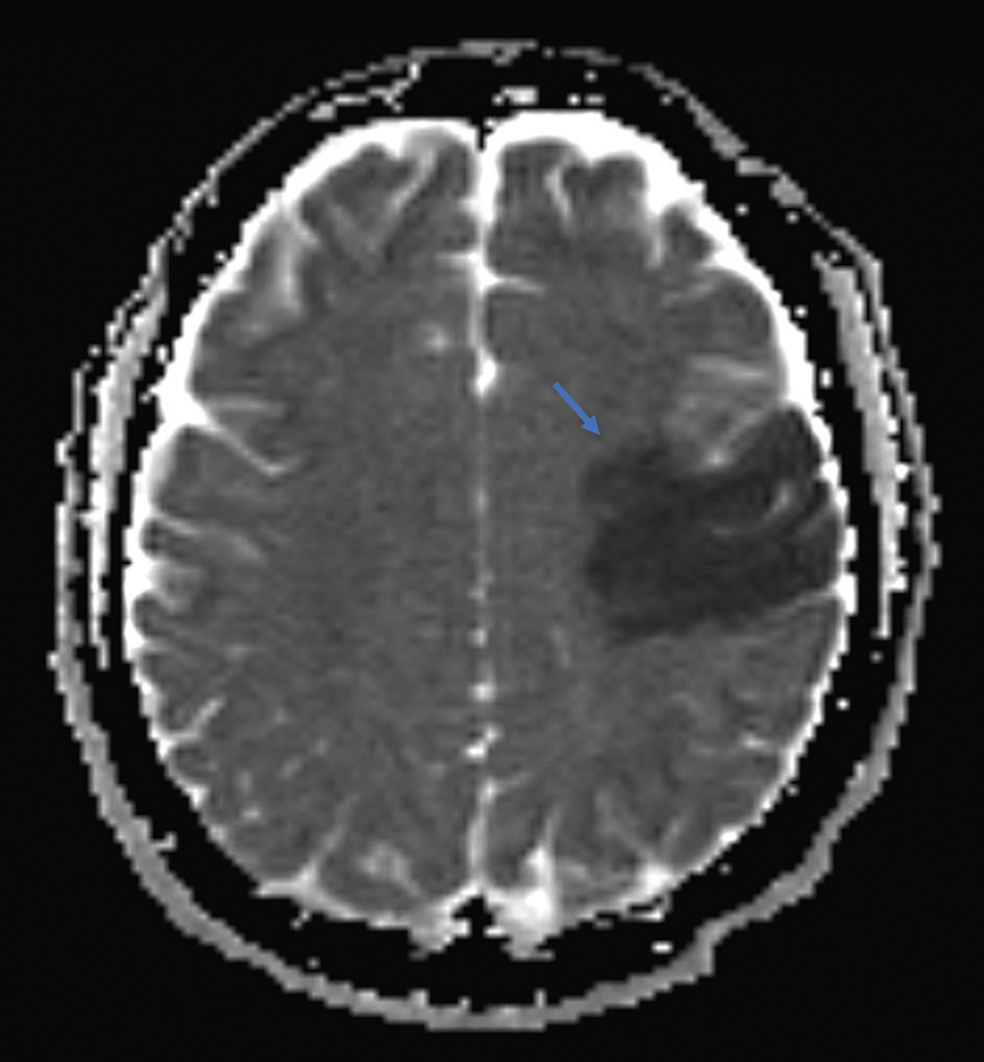
Evolving acute infarct in the left MCA territory as evidenced by the low ADC signal (Blue Arrow). MCA: middle cerebral artery; ADC: apparent diffusion coefficient

On May 15, 2021, the patient had another acute ischemic stroke, as shown in the MRI seen in Figure 2. He developed worsening stroke symptoms including right upper extremity weakness, right lower facial paresis, and expressive aphasia. He was found to have an acute evolving infarct of the left middle cerebral artery (MCA) distribution and smaller cortical infarcts without hemorrhagic transformation on full anticoagulation with heparin. Partial thromboplastin time (PTT) was measured at 79 seconds. The patient was not a candidate for repeat tPA administration or surgical thrombectomy given the recent tPA administration and the large area of infarct (after discussion with the local interventional stroke center).

Due to concern that the cause of the stroke was likely caused by an embolus from a ventricular thrombus in the setting of new onset dilated cardiomyopathy, the patient was continued on anticoagulation with heparin. He was transitioned from heparin to warfarin after an appropriate bridge. The patient was also evaluated and treated by physical therapy, occupational therapy, and speech-language pathology. The patient was able to make some progress with mild improvements in his neurologic deficits resulting from the second stroke including the ability to move his right leg and some improvement in right arm weakness. The patient was subsequently discharged from the ICU to the Veterans Affairs Hospital for continued acute stroke care.

## Discussion

COVID-19 typically presents as a viral respiratory disease, with patients developing fevers, chills, sore throat, and dyspnea. A subset of SARS-CoV-2 infections can present with neurological manifestations such as anosmia, ageusia, memory issues, difficulty with concentrating, and ataxia [5]. Severe complications of this disease can result in ARDS, pulmonary embolism, ischemic stroke, and myocarditis. The incidence of acute ischemic stroke in hospitalized patients with COVID-19 is approximately 2-3% and the incidence increases in those patients that are admitted to the ICU [6].

A correlation between COVID-19 infection and hypercoagulability has been demonstrated, but the mechanism behind this phenomenon is not fully understood. The hypercoagulability seen in COVID-19 patients can be examined through the lens of Virchow’s triad of endothelial injury, venous stasis, and hypercoagulable state. In vitro studies have shown evidence of direct invasion of endothelial cells by the SARS-CoV-2 virus resulting in cell injury and microvascular inflammation [7]. Another proposed mechanism of endothelial injury is associated with a systemic inflammatory response resulting in the upregulation of cytokines such as interleukin 6 (IL-6) and other acute-phase reactants [8]. Severely ill patients with COVID-19 have an increased risk of prolonged immobilization that can result in venous stasis during their course of hospitalization. This patient was also diagnosed with acute dilated cardiomyopathy secondary to myocarditis. It was deduced that the cardiomyopathy developed as a result of COVID-19 as the patient did not have any other risk factors such as recent surgery, prolonged immobilization, flight travel, hormone use, or previous thrombotic events. Cardiomyopathy has been shown to increase the risk of developing left ventricular thrombi, which can lead to thromboembolic events [9]. The hypercoagulable state observed in COVID-19 patients has been linked to increased circulation of prothrombotic factors. Studies have shown elevated factor VIII levels, fibrinogen, prothrombotic microparticles, neutrophil extracellular traps, and hyperviscosity [10-12]. Given the risk of hypercoagulability in COVID-19 patients, it is important to maintain a high index of suspicion for thrombotic events such as ischemic stroke, deep venous thrombosis, and pulmonary embolism.

The mainstay of prevention for these disease processes is anticoagulation. The current CDC guidelines recommend prophylactic dose low molecular weight heparin or unfractionated heparin to prevent venous thromboembolism in all COVID-19 patients in the inpatient setting. For critically ill patients admitted to the ICU or those with increasing oxygen requirements, the current recommendation is a therapeutic dose of heparin as measured by PTT between 60 and 100 [13]. Heparin is preferred over oral anticoagulation due to its short half-life and ease of reversibility. The CDC recommends against routine prophylactic anticoagulation for non-hospitalized patients without evidence of venous or arterial thromboembolism [13]. Our patient was started on a therapeutic dose of heparin and was then subsequently bridged to warfarin.

## Conclusions

Acute ischemic stroke in a young patient without significant risk factors or comorbidities is unusual. COVID-19 has been shown to directly increase the risk of hypercoagulability. Our patient is a healthy 29-year-old male without any significant risk factors for developing an acute ischemic stroke. The likely cause of the thrombotic event in our patient is hypercoagulability due to COVID 19 and stasis of blood resulting from acute dilated cardiomyopathy that developed due to myocarditis. SARS-COV-2 has many variable presentations and complications. It is important to understand the associated morbidity and mortality to guide patients towards favorable outcomes. Prophylactic anticoagulation against venous thrombosis in COVID 19 patients admitted to the hospital is strongly recommended by the CDC.
